# Relationship between season, lactation number and incidence of clinical mastitis in different stages of lactation in a Holstein dairy farm

**Published:** 2014

**Authors:** Maede Moosavi, Abdolah Mirzaei, Mohsen Ghavami, Amin Tamadon

**Affiliations:** 1 *Department of Clinical Sciences, School of Veterinary Medicine, Shiraz University, Shiraz, Iran; *; 2 *Private Veterinary practitioner, Nemoneh Dairy Farm of Astan-e-Ghods, Mashhad, Iran; *; 3 *Transgenic Technology Research Center, Shiraz University of Medical Sciences, Shiraz, Iran.*

**Keywords:** Clinical mastitis, Dairy cow, Lactation, Season

## Abstract

The aim of the present study was to compare the occurrence and duration of clinical mastitis in different seasons, stages of lactation period and parities in a Holstein dairy farm in Iran. A retrospective epidemiological survey from April 2005 to March 2008 was conducted on 884 clinical mastitis cases of 7437 lactations. Data of each case including calendar-date of mastitis onset, days in milk (DIM) of mastitis onset (early: 0-74 DIM; middle: 75-150 DIM, and late ≥ 150 DIM), duration of mastitis, and parity (1, 2, and ≥ 3) were recorded. Based on date of mastitis onset, cases were classified into stages of lactation. Moreover, beginning of mastitis was seasonally categorized. Duration of clinical mastitis after treatment in early lactation was less than late lactation in the first-parity cows (*p* = 0.005). In early lactation period, the first-parity cows suffered clinical mastitis in days earlier than two other parity groups (*p* < 0.001). Moreover, in late lactation period, the first-parity cows had clinical mastitis in days later than cows in the third and more parities (*p* = 0.002). Occurrence of clinical mastitis in summer increased in late lactation period but in winter increased in early lactation period (*p* = 0.001). In addition, occurrence time of clinical mastitis in summer were in days later than in spring (*p* = 0.02) and winter (*p* = 0.03) in early lactation period. In conclusion, occurrence of mastitis in winter and spring during early lactation and in summer during late lactation period were more prevalent especially in lower parities.

## Introduction

Clinical mastitis is one of the most costly multifactorial diseases in dairy farms which can be controlled by detection of predisposing factors. Clinical mastitis in cow is defined with signs such as inflammation of mammary glands with physical, chemical and microbiological changes.^1^

Many factors influence the incidence of clinical mastitis. Production stages of a cow,^2^^,^^3^ lactation number,^3^^-^^5^ herd management,^6^^,^^7^ and environment^2^ can be the major risk factors of clinical mastitis. Another studies investigated the association between incidence rate of clinical mastitis and clinical mastitis history,^8^ temperature, humidity and season (climatologic factors).^2^^,^^9^ In addition, associations between season, month in lactation, parity, and incidence rate of clinical mastitis have been shown.^2^^,^^10^ A recent study reported the effect of cow-specific risk factors such as parity, lactation month, and season of calving on the clinical mastitis of dairy cows from 15 days before to 120 days after calving in Iran.^11^

Understanding the seasonal and days in milk (DIM, stages of lactation period) patterns of clinical mastitis will improve disease prevention and control in dairy herds. The aims of the present study were 1) to determine the relationship between occurrence of clinical mastitis and parity during the various stages of lactation period, 2) to compare the occurrence of clinical mastitis in all seasons, and 3) to compare the mastitis duration following treatment of cow with different parity in a Holstein dairy farm.

## Materials and Methods

A retrospective epidemiological survey from April 2005 to March 2008 was conducted using data from a management program in a high-producing Holstein-Friesian dairy herd in Mashhad (latitude of 36° 20′ N and longitude 59° 35′ E, 980 m above the sea level) the northeast of Iran. The data series covered 7437 lactations. Mean lactation number was 2.16 and ranged from 1 to 11 lactations. Throughout the year, the cows were kept under roofed structures (free-stall barns) with open sides (zero-grazing system) and washed sand for bedding. They were grouped according to their milk production and fed according to the NRC 2001.^12^ The ration (total mixed ration) included mainly alfalfa, corn silage, beet pulp, cotton seed, soybean, corn, and barley. The cows under study were non-seasonal with year-round calving. The cows were machine-milked three times daily. The mean peak milk yield (90 days in milk) of the cows was 56 kg per day. The mean size of the herd was 1542 cows during the period of study. Dry cows were kept in a separate group and transferred three weeks prior to parturition to a close-up group. All animals were tested free of tuberculosis and brucellosis. The voluntary waiting period from calving to the first artificial insemination established for this dairy herd was 45 days. All cows were artificially inseminated. Mastitis data considered here were clinical mastitis cases (n = 884) detected by a veterinary clinician at milking time based on the presence of clots in the milk or during the dry period on the basis of clinical signs such as a hard or swollen udder. Mastitis cases were systematically treated with antibiotics that were administered topically to the udder or by systemic administration for cows with the most severe cases.

**Fig. 1:**
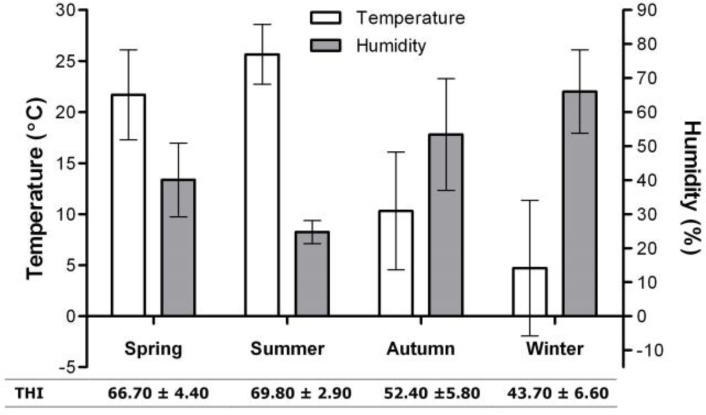
Mean ± SD of temperature, humidity and temperature-humidity index (THI) alterations in dairy farm during the period of study (from April 2005 to March 2008).

Data of each case including calendar-date of mastitis onset, occurrence day of clinical mastitis, duration (days) of mastitis (based on the clinical signs and cure), and parity (1, 2, and ≥ 3) were recorded. Based on the date of mastitis onset, cases were categorized into early (0-74 days in milk, DIM), mid (75-150 DIM), and late (≥ 150 DIM) stages of lactation. Moreover, onsets of mastitis were seasonally categorized. In the geographical area of our study there are four clearly distinguishable seasons. Climatological information regarding this location during the course of the study is summarized in Figure 1. Temperature-humidity index (THI) was calculated: 

*THI= (1.8* *×* *T + 32) – [(0.55 – 0.0055* *×* *RH)* *×* *(1.8* *×* *T–26)]*

where, T = Temperature and RH=Relative Humidity.

Mean (± SD) of clinical mastitis duration at different stage of lactation and occurrence day of clinical mastitis onset at different parity or different season were statistically compared using one-way ANOVA (Version 11.5; SPSS Inc., Chicago, Illinois). Least significant difference (LSD) post hoc test was used for comparison of means within the groups. Proportion of clinical mastitis cases of different stage of lactation period in different parity were statistically analyzed with the application of the Chi-square test. The coarse curve was used to show general trend of onset of mastitis occurrence during the lactation period using the fit spline option of GraphPad Prism (Version 5.01; GraphPad software Inc., San Diego, CA, USA). Values of *p* ≤ 0.05 were considered significant.

## Results

**Fig. 2:**
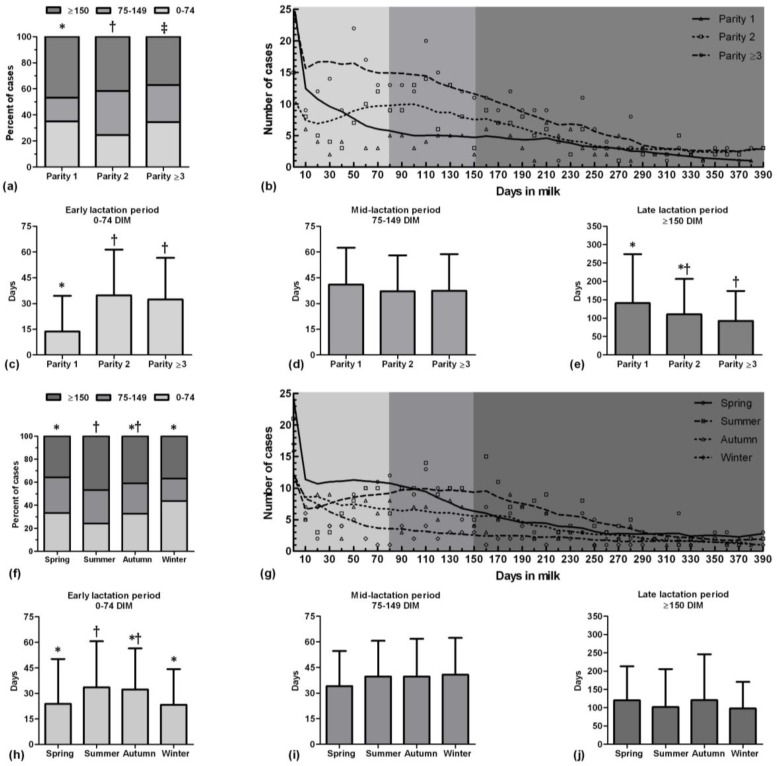
Proportion of percent of clinical mastitis cases of different stage of lactation period in different parity (a) and different seasons (f) of Holstein dairy cows; Locally weighted scatterplot smoothing (Lowess) curves of clinical mastitis cases which show the general trend of clinical mastitis incidence during a lactation period in different parity (b) and different season (g); Occurrence time (day) of clinical mastitis in different parity (c, d, and e) and different season (h, i, and j) of each lactation period. Different symbols show significant different between bars (*p* < 0.05).

Mean incidence rate of clinical mastitis was 30% during the period of study. Duration of clinical mastitis after treatment in early lactation (3.57 ± 2.45 days) was less than late lactation (4.95 ± 2.99 days) in the first-parity cows (*p* = 0.005). However, there was no difference in duration of clinical mastitis after treatment between lactation periods of cows in the other parity. In addition, the clinical mastitis occurrence at each lactation period was different between cows in the first, second and more than the second-parities (*p* = 0.002), (Figs. 2a and 2b). In early lactation period, the first-parity cows suffered clinical mastitis in earlier day than two other parity groups (*p* < 0.001), (Fig. 2c). However, occurrence time (day) of clinical mastitis was not different between three groups of parity in mid lactation period (*p* > 0.05), (Fig. 2d). Moreover, the first-parity cows showed clinical mastitis in later day than cows in the third and more parity in late lactation period (*p* = 0.002), (Fig. 2e).

Occurrence of clinical mastitis in summer increased in late lactation period but in winter increased in early lactation period (*p* = 0.001), (Figs. 2f and 2g). In addition, occurrence time of clinical mastitis in summer were later than in spring (*p* = 0.02) and winter (*p* = 0.03) in early lactation period (Fig. 2h). However, occurrence time of clinical mastitis was not different between four seasons in mid and late lactation periods (*p* > 0.05), (Figs 2i and 2j).

**Fig. 3:**
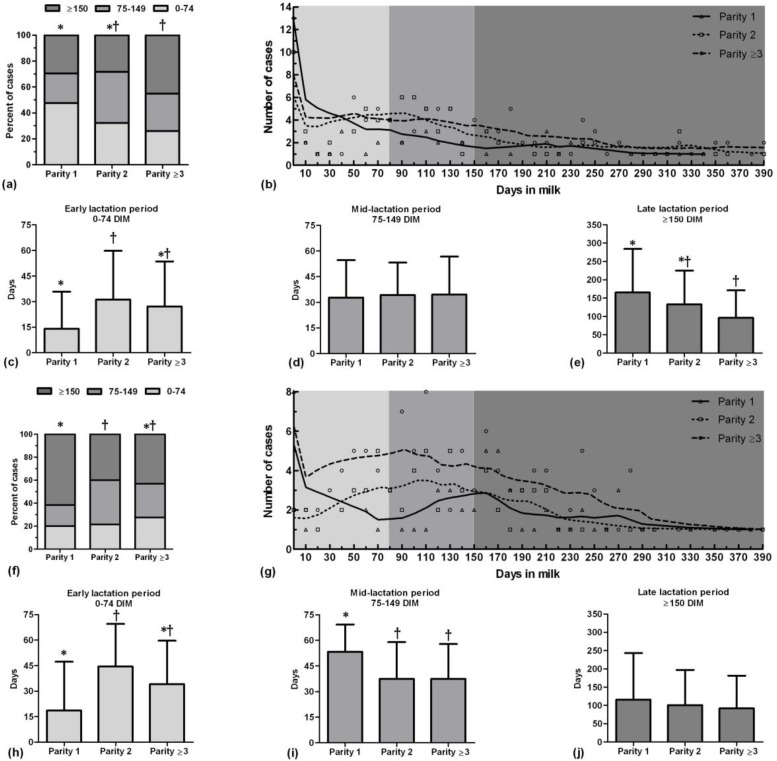
Proportion of percent of clinical mastitis cases of different stage of lactation period in different parities of Holstein dairy cows in spring (a) and summer (f); Lowess curves of clinical mastitis cases which show the general trend of clinical mastitis incidence during a lactation period in different parity in spring (b) and summer (g); Occurrence time (day) of clinical mastitis in different parity of each lactation period in spring (c, d, and e) and summer (h, i, and j). Different symbols show significant different between bars (*p* < 0.05).

In spring, occurrence of clinical mastitis in the first-parity cows increased in early lactation period and in the third and more parity cows increased in late lactation period (*p* = 0.02), (Figs. 3a and 3b). In early lactation period, the first-parity cows suffered clinical mastitis in the earlier days than the second-parity cows (*p* = 0.01), (Fig. 3c). However, occurrence time of clinical mastitis was not different between three groups of parity in mid lactation period (*p* > 0.05), (Fig. 3d). Moreover, the first-parity cows showed clinical mastitis in later day than cows in the third and more parity in late lactation period (*p* = 0.02), (Fig. 3e).

In summer, occurrence of clinical mastitis in the first-parity cows increased in late lactation period and in the second-parity cows increased in mid lactation period (*p* = 0.02, Figs. 3f and 3g). In early lactation period, the first-parity cows suffered clinical mastitis in earlier day than the second-parity cows (*p* = 0.01), (Fig. 3h). Moreover, the first-parity cows showed clinical mastitis in later day than two other parity groups in mid lactation period (*p* < 0.05), (Fig. 3i). However, occurrence time of clinical mastitis was not different between three groups of parity in late lactation period (*p* > 0.05), (Fig. 3j).

**Fig. 4:**
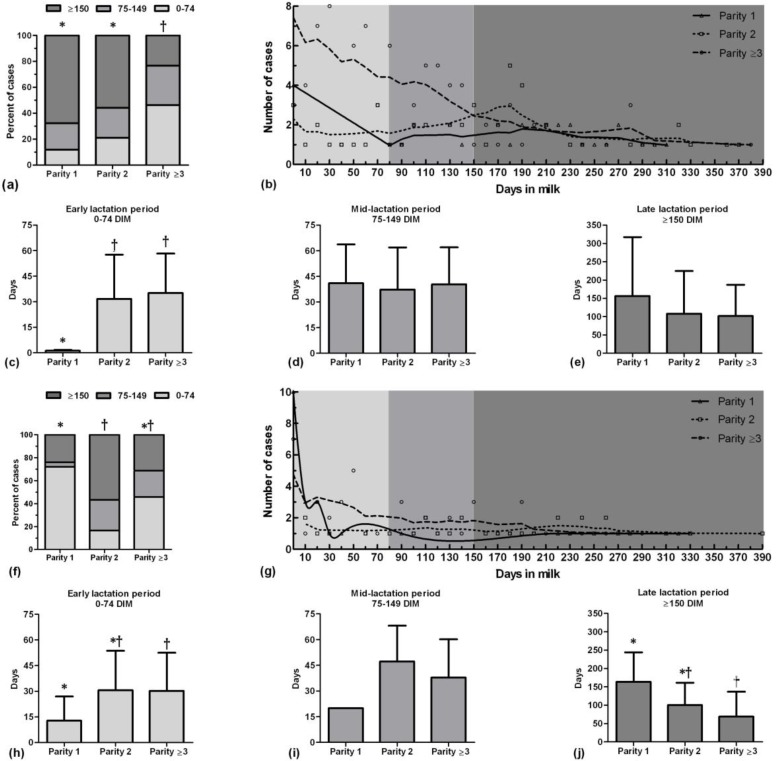
Proportion of percent of clinical mastitis cases of different stage of lactation period in different parity of Holstein dairy cows in autumn (a) and winter (f); Lowess curves of clinical mastitis cases which show the general trend of clinical mastitis incidence during a lactation period in different parity in autumn (b) and winter (g); Occurrence time (day) of clinical mastitis in different parity of each lactation period in autumn (c, d, and e) and winter (h, i, and j). Different symbols show significant different between bars (*p* < 0.05).

In autumn, occurrence of clinical mastitis in the first and second parity cows increased in late lactation period and in the third and more parity cows increased in early lactation period (*p* < 0.05), (Figs. 4a and 4b). In early lactation period, the first-parity cows suffered clinical mastitis in the earlier day than two other parity groups (*p* < 0.05), (Fig. 4c). However, occurrence time of clinical mastitis was not different between three groups of parity in mid and late lactation period (*p* > 0.05), (Figs. 4d and 4e). 

In winter, occurrence of clinical mastitis in the first-parity cows increased in early lactation period, in the second-parity cows increased in late lactation period (*p* < 0.001), (Figs. 4f and 4g). In early lactation period, the first-parity cows suffered clinical mastitis in earlier day than the third and more parity cows (*p* = 0.02), (Fig. 4h). However, occurrence time of clinical mastitis was not different between three groups of parity in mid lactation period (*p* > 0.05), (Fig. 4i). Moreover, the first-parity cows showed clinical mastitis in later day than cows in the third and more parity in late lactation period (*p* = 0.006), (Fig. 4j).

## Discussion

In primiparous cows, duration of clinical mastitis in early lactation was less than late lactation. However, in multiparous cows there was no difference in duration of clinical mastitis between different stages of lactation period. In early lactation period, primiparous cows suffered clinical mastitis in earlier time than multiparous. However in late lactation, clinical mastitis occurred later in primiparous than multiparous cows. Mungube *et al*. reported the significant association between clinical mastitis and cows with at least eight parities and cows in at least the fourth month of lactation period.^   13 ^ The risk of having clinical mastitis was significantly influenced by lactation month and parity of cow.^   10 ^ Multiparous cows had higher incidence of clinical mastitis compared with heifers over the all stages of lactation period.^   2 ^ Some of researchers reported that cows in early lactation period or in the first month of lactation and cows in higher parities were associated with an increased clinical mastitis.^3^^,^^13^^,^^14^  Clinical mastitis most often happens around parturition.  ^15^^-^^18^  Health of the mammary gland is at high risk during two weeks prior and after calving.^   19^

Hormonal changes include increasing a five-fold of blood cortisol concentration and 17 β-oestradiol levels occur at the day of parturition.^20^^-^^22^ Thus, the immune system of dairy cows was suppressed during the periparturient period.^23^^,^^24^ Consequently, the cross-talk between neuro-endocrine and immune system of cows during the peri-parturient period is associated with the higher incidence of sever clinical mastitis.^   25 ^ Thus, response to treatment may be different in primiparous mastitic cows with various stage of lactation but it was similar for multiparous cows. It can be concluded that the most important stage of lactation in high risk for mastitis occurrence depends on the parity of cows. The early lactation is critical stage for primiparous cows in winter and spring while it was important for the third parity in winter and autumn. The late lactation is critical stage for primiparous cows in summer and autumn while it was important for the second-parity in autumn and winter but in spring for the third parity.

In spring and winter, occurrence of clinical mastitis in primiparous cows increased in early lactation period and in multiparous cows increased in late lactation period. The high percentage of humidity in both seasons may cause the most growth of pathogenic agents. Some deficiencies such as selenium and vitamin E deficiency result in an increased incidence of clinical mastitis and dietary supplementation improves udder health, with the effects most evident at calving and early lactation.^   26 ^ These deficiencies can routinely happen during late pregnancy and early lactation. Susceptibility to infection in primiparous cows may due to unexpected calving, especially on the ground. Moreover, udder edema mostly occurs at their calving, that is commonly observed during late pregnancy and early lactation. In cows with udder edema, milking is a painful process and milk let-down is poor. Consequently, the ability of the teat and udder tissues to resist bacterial challenge during the calving period is diminished and susceptibility to infection is increased.^   27 ^ Many over-conditioned primiparous cows had a higher prevalence of mastitis due to release of milk drops from their teats prior to calving.^   28 ^

In summer, immunosuppression because of heat stress (THI in summer > 72) may also have a role for higher occurrence rate of clinical mastitis in primiparous cows during late lactation.^   2 ^ The mastitic cows in spring and winter had lower days in milk after calving compared to that of cows in summer during early lactation period. Ghavi Hossein-Zadeh and Ardalan reported the odds of clinical mastitis increased in multiparous cows (odds ratio, OR = 2.83), in winter season (OR = 1.68) and in the first month of lactation (OR = 3.38).^   11 ^ Clinical mastitis happened in late fall more than in the summer.^   2 ^ Cows were in high risk for clinical mastitis in summer more than in winter.^   2^

In conclusion, it seems important to focus on diagnosis and treatment of mastitis in winter and spring during early lactation period and in summer during late lactation period. However, in other situations such as other seasons, parity and stage of lactation it is also important to consider the diagnosis and treatment of mastitis to achieve the best strategy for the treatment and prevention of mastitis in dairy cows. Thus, the present study demonstrates the importance of the mastitis control program as a herd management practice. Specific recommendations to the dairy cow farmers for control of mastitis and training programs for farm managers and staff can improve udder health status based on the incidence rate of mastitis in dairy cows with different parity during various stages of lactation period in four distinct seasons in dairy herds.

## References

[1] Halasa T, Huijps K, Østerås O (2007). Economic effects of bovine mastitis and mastitis management: A review. Vet Q.

[2] Olde Riekerink RGM, Barkema HW, Stryhn H (2007). The effect of season on somatic cell count and the incidence of clinical mastitis. J Dairy Sci.

[3] Suriyasathaporn W, Schukken YH, Nielen M (2000). Low somatic cell count: A risk factor for subsequent clinical mastitis in a dairy herd. J Dairy Sci.

[4] Beaudeau F, Seegers H, Fourichon C (1998). Association between milk somatic cell counts up to 400,000 cells/ ml and clinical mastitis in French Holstein cows. Vet Rec.

[5] Faye B, Perochon L, Dorr N (1998). Relationship between individual-cow udder health status in early lactation and dairy cow characteristics in Brittany, France. Vet Res.

[6] Barkema HW, Schukken YH, Lam TJGM (1999). Manage-ment practices associated with the incidence rate of clinical mastitis. J Dairy Sci.

[7] Nyman AK, Ekman T, Emanuelson U (2007). Risk factors associated with the incidence of veterinary-treated clinical mastitis in Swedish dairy herds with a high milk yield and a low prevalence of subclinical mastitis. Prev Vet Med.

[8] Houben EHP, Dijkhuizen AA, Van Arendonk JAM (1993). Short- and long-term production losses and repeatability of clinical mastitis in dairy cattle. J Dairy Sci.

[9] Morse D, DeLorenzo MA, Wilcox CJ (1988). Climatic effects on occurrence of clinical mastitis. J Dairy Sci.

[10] Steeneveld W, Hogeveen H, Barkema HW (2008). The influence of cow factors on the incidence of clinical mastitis in dairy cows. J Dairy Sci.

[11] Ghavi Hossein-Zadeh N, Ardalan M (2011). Cow-specific risk factors for retained placenta, metritis and clinical mastitis in Holstein cows. Vet Res Commun.

[12] NRC (National Research Council) (2001). Nutrient requirements of dairy cattle.

[13] Mungube EO, Tenhagen BA, Kassa T (2004). Risk factors for dairy cow mastitis in the central highlands of Ethiopia. Trop Anim Health Prod.

[14] Kerro Dego O, Tareke F (2003). Bovine mastitis in selected areas of southern Ethiopia. Trop Anim Health Prod.

[15] Bradley AJ, Green MJ (2004). The importance of the non-lactating period in the epidemiology of intramammary infection and strategies for prevention. Vet Clin North Am Food Anim Pract.

[16] McDougall S, Arthur DG, Bryan MA (2007). Clinical and bacteriological response to treatment of clinical mastitis with one of three intramammary antibiotics. N Z Vet J.

[17] Svensson C, Nyman AK, Waller KP (2006). Effects of housing, management, and health of dairy heifers on first-lactation udder health in southwest Sweden. J Dairy Sci.

[18] Valde JP, Lawson LG, Lindberg A (2004). Cumulative risk of bovine mastitis treatments in Denmark, Finland, Norway and Sweden. Acta Vet Scand.

[19] Oliver SP, Sordillo LM (1988). Udder health in the peri-parturient period. J Dairy Sci.

[20] Burton JL, Madsen SA, Chang LC (2005). Gene expression signatures in neutrophils exposed to glucocorticoids: A new paradigm to help explain "neutrophil dysfunction" in parturient dairy cows. Vet Immunol Immunopathol.

[21] Lamote I, Meyer E, Duchateau L (2004). Influence of 17 β-estradiol, progesterone, and dexamethasone on diapedesis and viability of bovine blood polymorpho-nuclear leukocytes. J Dairy Sci.

[22] Weber PSD, Toelboell T, Chang LC (2004). Mechanisms of glucocorticoid-induced down-regulation of neutrophil L-selectin in cattle: Evidence for effects at the gene-expression level and primarily on blood neutrophils. J Leukoc Biol.

[23] Lippolis JD, Reinhardt TA, Goff JP (2006). Neutrophil extracellular trap formation by bovine neutrophils is not inhibited by milk. Vet Immunol Immunopathol.

[24] Pyörälä S (2008). Mastitis in post-partum dairy cows. Reprod Domest Anim.

[25] Vangroenweghe F, Lamote I, Burvenich C (2005). Physiology of the periparturient period and its relation to severity of clinical mastitis. Domest Anim Endocrinol.

[26] Weiss WP, Hogan JS, Smith KL (1990). Relationships among selenium, vitamin E, and mammary gland health in commercial dairy herds. J Dairy Sci.

[27] Slettbakk T, Jorstad A, Farver TB (1995). Impact of milking characteristics and morphology of udder and teats on clinical mastitis in first-and second-lactation Norwegian cattle. Prev Vet Med.

[28] Pankey JW, Pankey PB, Barker RM (1996). The prevalence of mastitis in primiparous heifers in eleven Waikato dairy herds. N Z Vet J.

